# Evaluation of a Tetracycline-Resistant *E. coli* Enumeration Method for Correctly Classifying *E. coli* in Environmental Waters in Kentucky, USA

**DOI:** 10.3390/pathogens12091090

**Published:** 2023-08-28

**Authors:** Callie Boggs, Kidus Shiferawe, Eckhardt Karsten, Jayden Hamlet, S. Travis Altheide, Jason W. Marion

**Affiliations:** 1 Environmental Health Science and Sustainability Program, Eastern Kentucky University, Richmond, KY 40475, USA; callie_boggs30@mymail.eku.edu (C.B.); semekidus_shifera@mymail.eku.edu (K.S.); 2 Department of Microbiology, Miami University, Oxford, OH 45042, USA; karsteea@miamioh.edu; 3 School of Natural Sciences and Mathematics, Stockton University, Galloway, NJ 08205, USA; hamletj1@go.stockton.edu; 4 Medical Laboratory Science Program, Eastern Kentucky University, Richmond, KY 40475, USA; travis.altheide@eku.edu; 5 Eastern Scientific LLC, Richmond, KY 40475, USA

**Keywords:** *Escherichia coli*, *Enterobacter cloacae*, tetracycline resistance, one health, water quality, antimicrobial resistance, antibiotic resistance, *Enterobacter*, culturable, monitoring

## Abstract

The global concern over antimicrobial resistance (AMR) and its impact on human health is evident, with approximately 4.95 million annual deaths attributed to antibiotic resistance. Regions with inadequate water, sanitation, and hygiene face challenges in responding to AMR threats. Enteric bacteria, particularly *E. coli*, are common agents linked to AMR-related deaths (23% of cases). Culture-based methods for detecting tetracycline-resistant *E. coli* may be of practical value for AMR monitoring in limited resource environments. This study evaluated the ColiGlow™ method with tetracycline for classifying tetracycline-resistant *E. coli*. A total of 61 surface water samples from Kentucky, USA (2020–2022), provided 61 presumed *E. coli* isolates, of which 28 isolates were obtained from tetracycline-treated media. Species identification and tetracycline resistance evaluation were performed. It was found that 82% of isolates were *E. coli*, and 18% were other species; 97% were identified as *E. coli* when using the API20E identification system. The MicroScan system yielded *Enterobacter cloacae* false positives in 20% of isolates. Adding tetracycline to ColiGlow increased the odds of isolating tetracycline-resistant *E. coli* 18-fold. Tetracycline-treated samples yielded 100% tetracycline-resistant *E. coli* when the total *E. coli* densities were within the enumeration range of the method. ColiGlow with tetracycline shows promise for monitoring tetracycline-resistant *E. coli* in natural waters and potentially aiding AMR surveillance in resource-limited settings among other environments.

## 1. Introduction

Human health risks related to antimicrobial resistance (AMR) are a great global concern with a recent estimate associating 4.95 million deaths in 2019 to antibiotic resistance [1]. A recent assessment from the U.S. Centers for Disease Control and Prevention (CDC) described that the U.S. has had 35,000 deaths from 2.8 million infections from antibiotic-resistant bacteria [2]. While progress reducing the prevalence of AMR infections and mortality has been made in the U.S. in the last decade [2], many nations and regions are having trouble responding to the threat posed [3]. The AMR threat is particularly greater in regions with inadequate water, sanitation, and hygiene [1,4] as evidenced in a recent metagenomic analysis of 1589 fecal metagenomes which demonstrated significantly higher abundances of antimicrobial resistance genes (ARGs) in samples from nations lacking improved water and sanitation [5]. 

In estimating the most common pathogens linked to AMR-related deaths globally, the enteric bacteria *E. coli* and *Klebsiella pneumoniae* represent the top and third most common agents, respectively [5], in which *E. coli* have been implicated in 23% of the total deaths from antibiotic resistance [1]. The emergence and spread of resistant microbiota or genes from regions lacking improved water and sanitation to clinical environments has already happened [6]. Accordingly, increased densities of resistant microbes are greater in areas needing improvements to water, sanitation, and hygiene systems since these areas experience more waterborne and foodborne illnesses, and thus use more antimicrobial agents for patient and animal care [1,4]. While recommendations for antimicrobial stewardship are promoted, the scientific community advises environmental monitoring of AMR to inform data-driven approaches for guiding interventions [1,6,7,8] through a One Health framework [9,10,11].

The National Antimicrobial Resistance Monitoring System (NARMS) strategic plan for the U.S. recommends that surveillance should begin with surface waters since they integrate differentially affected ecosystems [8]. For a potential target organism to study for enteric bacteria resistance, *E. coli* may be a good starting point since existing water quality monitoring guidelines for freshwater recreation/bathing and drinking already use *E. coli* [12,13,14].

The focus of this study is assessing the validity of culture-based method results for tetracycline-resistant *E. coli,* while recognizing molecular approaches in general are preferred for future methodological monitoring standards [15]. Among these methods, qPCR is one expected to be among future gold-standard molecular methods for quantifying fecal indicator markers and pathogens [16]. Regarding qPCR, while tremendous progress has been made in reducing equipment, software, and consumable costs for fecal indicators and pathogens in water, molecular methods including qPCR present major obstacles for limited resource environments including high costs, specialized instruments, and training/personnel costs [16]. Thus, culture-based methods have some advantages in resource-limited environments with respect to the 20 characteristics identified by Bain et al. [17]. For low-resource settings, interest remains in new approaches for detecting *E. coli* and quantitatively assessing *E. coli* density even when results take 24 h [18], and this logic may hold true for AMR monitoring.

In generating a framework for antibiotic resistance monitoring in the water environment, *int1*, *blaCTX-M*, *sul1*, *vanA*, and *tet*(A), have been identified as valuable molecular targets [15]. Using these genes to inform culture-based methods, beta-lactams, sulfonamides, vancomycin, and tetracyclines would be potential media additives for assessing resistance. While all candidates have stability in spiked river samples after six days [19], beta-lactams have been reported to degrade in growth media [20]. Among the others, tetracycline stability in the environment has been more frequently raised as a point of concern in the literature [21] and tetracycline resistance among *E. coli* is common globally [22]. 

Based upon a presumed monitoring need for limited resource environments, the purpose of this study was to evaluate the classification ability of the ColiGlow™ *E. coli* enumeration method [23] to properly classify *E. coli* species identification and antibiotic resistance status upon the addition of an antibiotic. Tetracycline was selected for evaluation as a recent study from an adjacent U.S. state (Ohio) demonstrated that tetracycline resistance would likely be observed in natural waters in the U.S., as has been observed in 8.8% of 329 *E. coli* isolates from the Maumee River (Ohio, USA) and 27% of over 200 isolates from prairie, cropland, and hay pasture runoff in Texas, USA [24,25]. Findings from this study may have application to culture-based methods for practically assessing the presence/absence or density of tetracycline-resistant culturable *E. coli* with improved efficiency and lower costs than existing multi-step methods.

## 2. Materials and Methods

### 2.1. Study Area and Sample Collection 

Surface water samples (n = 61) used for *E. coli* isolate analysis were collected in Kentucky, USA, during 2020, 2021, and 2022 from a diverse array of surface water types. The 61 samples are a portion of 112 natural water samples that were analyzed including 112 paired tests, whereby 112 tests were performed with tetracycline-treated media, and 112 tests were performed using media without the tetracycline antibiotic. All water samples were collected in sterile Whirl-Pak^®^ bags and processed at Eastern Kentucky University (Richmond, Kentucky, USA) within six hours of collection to facilitate *E. coli* growth using an *E. coli* enumeration method (ColiGlow™) in a 96-well plate format for each sample [23]. 

A total of 61 isolates were evaluated. These 61 isolates were obtained from a random selection of the 96-well ColiGlow plates presenting at least one fluorescing well, whereby a fluorescing well was hypothesized to contain culturable *E. coli*. For assessing whether these fluorescing wells contained culturable *E. coli*, the 61 isolates were obtained for the purpose of evaluating the correct or incorrect classification of the *E. coli* species identification and tetracycline susceptibility. 

To increase sample diversity and independence, only one isolate was obtained from each of these 61 selected ColiGlow plates. For plates with more than one fluorescing well, only one of the fluorescing wells in the 96-well plate was used to obtain an isolate. Therefore, 61 total isolates were obtained in this study which were randomly selected from 61 ColiGlow plates presenting presumed *E. coli* growth among the 224 total plates used. Given that ColiGlow plates containing tetracycline-treated samples had many samples with no growth (no fluorescing wells) there were fewer plates to randomly select for isolate evaluation. Ultimately, 33 isolates were obtained from 33 ColiGlow plates with no tetracycline in the media, and 28 isolates were obtained from 28 ColiGlow plates with tetracycline in the media. 

Among the 61 samples used for obtaining isolates, 20 samples were collected in 2020 from central Kentucky (Madison County) including 16 samples from free-flowing streams in a cattle-producing region, two samples from a lake, and two samples from roadside ditches associated with cow pasture runoff. In 2021, eleven samples were collected in central and southeast Kentucky with five samples from free-flowing streams impacted by surface mining for coal, five samples from free-flowing streams not impacted by mining, and one sample from a roadside ditch associated with cow pasture runoff. In 2022, 30 samples were collected in central and eastern Kentucky with 15 samples being free-flowing streams in heavily forested areas of the Daniel Boone National Forest and 15 samples being from a mixture of urban and residential free-flowing streams in central Kentucky. 

### 2.2. Obtaining Presumed E. coli and Tetracycline-Resistant E. coli Isolates

Each water sample was processed in accordance with a standard procedure for the ColiGlow test method. In brief, samples were processed in media with and without the tetracycline antibiotic. Specifically, 22.5 mL of the water sample was added to a 50 mL tube containing 2.5 mL of the liquid culture media for the selective differential growth of *E. coli.* Then, another 22.5 mL of the sample was added to a different tube containing the growth media plus either 320 µg (half tetracycline) or 640 µg of tetracycline (Fisher Scientific, Fair Lawn, New Jersey, USA, product code: BP912-100). The half tetracycline concentrations used were intended to increase test sensitivity while enabling the enumeration of *E. coli* with intermediate resistance.

**Figure 1:**
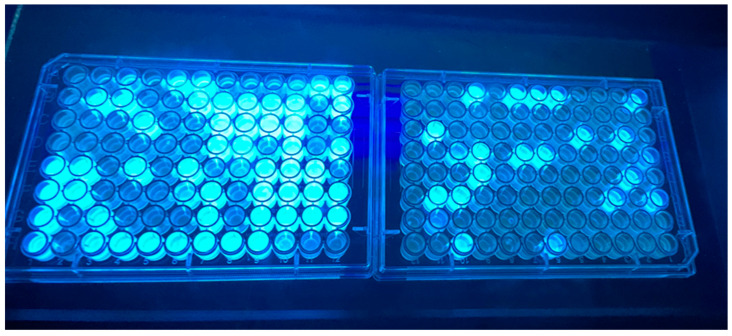
The results of one water sample evaluated for *E. coli* density by the ColiGlow method without tetracycline (**left**), and the ColiGlow method containing tetracycline (**right**) whereby the fluorescing wells under longwave ultraviolet light are indicative of presumed *E. coli* growth.

The sample mixtures were then inverted at least 30 times, poured into a multi-channel pipette reservoir, and then distributed into 96-well plates with a multi-channel pipette with 200 microliters being placed in each well. The prepared 96-well plates were covered and incubated at 35 °C for 24 h. Following incubation, the plates were viewed under a longwave ultraviolet light and fluorescing wells were counted as positive wells for presumed *E. coli* growth due to their β-D-glucuronidase enzymatic activity cleaving the 4-methylumbelliferyl-β-D-glucuronide (MUG) included in the growth media to produce the fluorogenic compound 4-methylumbelliferone (4-MU). Using the number of positive wells in conjunction with a most probable number (MPN) table provided with the ColiGlow test, the MPN was estimated per 100 mL of sample within a range. The range of the test kit is 14–1479 MPN per 100 mL for 1 glowing well to 95 glowing wells. A negative test was reported as <14 MPN per 100 mL and a test with 96 glowing wells was reported as overrange (>1479 MPN per 100 mL). An example of the ColiGlow method with fluorescing wells in the media with and without tetracycline is presented in Figure 1.

For obtaining isolates, a sterile 10 µL calibrated loop was dipped in one fluorescing well from a positive plate and then inoculated using a three-phase streak technique onto a modified membrane-Thermotolerant *E. coli* (modified mTEC) agar plate. This process was performed for the 61 selected plates with fluorescing wells. Following inoculation, the modified mTEC plates were incubated at 2 h for 35 °C and then 22 h at 44.5 °C in accordance with the incubation temperatures used in U.S. EPA Method 1603 [26]. Following incubation, the magenta-colored colonies were presumed to be *E. coli* [26]. The streak technique enabled the growth of many isolated colonies per plate, and among these colonies, one colony was selected at random from all the colonies present on each modified mTEC agar plate to be used for subsequent species identification and tetracycline resistance evaluation. If no magenta-colored isolate appeared following incubation on modified mTEC agar, then a randomly selected isolate from the colonies on the plate was used for subsequent evaluation of tetracycline resistance and species identification to discern which species was associated with the fluorescence in the 96-well plate. 

Similar research evaluating the specificity of Aquatest media with RUG™ for detecting, enumerating, and properly classifying *E. coli* used between one and five isolates per plate based upon colony morphology type [27]. In this study, only one isolate per sample (per mTEC plate) was used, as the laboratory resources required for subsequent antibiotic susceptibility analysis and species identification per isolate are substantial. Like similar research [27], isolates most likely to be *E. coli* were selected. 

### 2.3. Species Identification 

During 2020 and 2021, species identification was performed using API20E strips (bioMerieux, Marcy l’Etoile, France) in accordance with the manufacturer’s instructions using one API20E strip per isolate. In 2022, the university acquired new instrumentation allowing species identification to be performed using a Beckman Coulter MicroScan^®^ (Brea, CA, USA) with one Gram-negative Urine Combo 85 panel per isolate. The newer technology permitted simultaneous species identification and antibiotic susceptibility testing. 

Prior to inoculating the API20E strip and MicroScan panels, each isolate was inoculated from modified mTEC to blood agar and incubated for 18 h. A fresh isolate from blood agar was then inoculated into the API20E strip and MicroScan panels in accordance with the manufacturer’s instructions. 

Both identification methods (API20E and MicroScan) utilize a combination of biochemical tests during and after an 18 h incubation that generate color changes which provide a practical means for the identification of Gram-negative bacteria and members of the Enterobacteriaceae when compared against each manufacturer’s reference library. The MicroScan panels are advantageous over the API20E strips due to less human error and reliance on an automated microplate reader attached to PC and software [28,29]. The API20E strips rely upon an approach developed in the 1970s [28] that has been demonstrated recently to be error prone due to having a limited library of species and strains [29]. 

### 2.4. Assessment of Tetracycline Susceptibility/Resistance 

Assessment of tetracycline resistance occurred using minimum inhibitory concentration (MIC) methods. The MIC value obtained for each isolate in the presence of tetracycline was used to determine resistant isolates; whereby isolates with a tetracycline MIC > 16 µg/mL were deemed resistant, ≤4 µg/mL were susceptible, and the intermediate breakpoint was 8 µg/mL [30]. 

During 2020 and 2021, tetracycline MICs were obtained using tetracycline E-strip methods (bioMerieux, Marcy l’Etoile, France). Specifically, an inoculum (0.5 McFarland standard) was prepared for each isolate evaluated using the colonies from the same blood agar plates that were also used for species identification. Each inoculum was swabbed onto Mueller–Hinton agar to promote the growth of lawns. Immediately following plate inoculations, the E-strip containing a gradient of concentrations of tetracycline was placed on the media surface of each plate. The inoculated plates with the E-strips were then incubated for 24 h at 35 °C. Following incubation, MIC values were obtained by reading the printed concentration on the strip at the intersection with the zone of inhibition [31]. 

In 2022, tetracycline resistance was assessed using the MIC method that co-occurred with species identification procedures using a Beckman Coulter MicroScan^®^ (Brea, CA, USA) Urine Combo 85 panel. In addition to the biochemical tests, the Urine Combo 85 panel obtained the MIC values for several antibiotics including tetracycline. 

While two methods were used for tetracycline susceptibility testing, both methods presumably performed comparably. While comparisons of these methods for tetracycline susceptibility testing are not readily described in the literature, other comparisons exist. In the case of gentamicin susceptibility testing among Enterobacterales [32], Colistin resistance testing among *E. coli* [33], and ertapenem susceptibility among Enterobacteriaceae [34], the two methods used in this study provide similar results in those comparison studies.

### 2.5. Statistical Analysis

All data analyses were performed with Stata 14 [35]. Data analyses included summary statistics (mean, median, standard deviation) and cross-tabulations. Statistical tests included Chi-square and Fisher’s Exact tests for comparing frequency differences in the cross-tabulations; whereby Fisher’s Exact tests were used if any cell in the cross-tabulation had less than five observations. Logistic regression was used for obtaining odds ratios. Following logistic regression, model discrimination was assessed using the area under the ROC curve (AUC) [36]. 

## 3. Results and Discussion

### 3.1. Species Identification of Isolates

Among the 61 isolates investigated, 50 (82%) were identified as *E. coli* (Table 1). Among the eleven (18%) isolates not identified as *E. coli*, six were identified as *Enterobacter cloacae*. The other five isolates were identified as *Kluyvera ascorbata*, *Kluyvera intermedia*, *Klebsiella pneumoniae, Serratia odorifera*, and *Citrobacter braakii*. Variables potentially impacting species identification include the species identification method (API20E versus MicroScan) and the total density of *E. coli* and other bacteria in the sample associated with each isolate. 

Among the presumed *E. coli*-positive (fluorescing) wells from the ColiGlow plates, one isolate was obtained per well. Since only one isolate was obtained per well, the interpretation should also consider the possibility that *E. coli* may also have been present, but by chance was not picked as a colony from among the many isolated colonies that appeared on the modified mTEC agar plates following inoculation from the positive wells from the ColiGlow plates. As a study limitation, since only one colony was obtained from each modified mTEC plate, there was a possibility that *E. coli* were among the many colonies capable of growth in the media from the ColiGlow method and on modified mTEC agar. In similar research evaluating Aquatest containing the novel substrate RUG™, upon inoculating fluorescing wells on modified mTEC agar, numerous colonies were observable following incubation, and in their study, as many as five morphologies could be present [27]. In that study, one isolate of each colony morphology was evaluated. In this study evaluating ColiGlow, the most likely candidate isolate (magenta colony) was selected given the practical limitations of testing the tremendous diversity and number of all isolated colonies that were growing on the mTEC agar. 

For enhancing the likelihood of recovering *E. coli* isolates, using lessons learned from prior research [27], less differential media (MacConkey agar) was not used and modified mTEC agar was used, coupled with the elevated incubation temperature (44.5 °C) for obtaining thermotolerant isolates on the modified mTEC agar. This approach was consistent with studies evaluating Colilert-18™ [27,36] and the novel substrate RUG™ [27] as a means of isolating *E. coli*. Among all the isolates obtained in this study, only one (*C. braakii)* did not present magenta on the modified mTEC agar, but instead presented as a white colony with no magenta colonies present. In that case, it was presumed that the isolate was not likely *E. coli* as the modified mTEC agar colonies should present with magenta or red coloration to indicate β-D-glucuronidase enzymatic activity, which is the same enzyme associated with fluorescence in positive wells from the ColiGlow method as well as other *E. coli* identification/enumeration methodologies including Colilert-18 and AquaTest-RUG [27,37]. Specifically, the modified mTEC agar contains the chromagen 5-bromo-6-chloro-3-indolyl-β-D-glucuronide [26], which produces the magenta color in colonies exhibiting β-D-glucuronidase activity which catabolizes the compound [38,39]. 

It is noteworthy that while most (92% to 96%) *E. coli* from water have been reported to have β-D-glucuronidase activity within 24 h [40,41], there are some *E. coli* that do not express this enzyme and would likely not be detected by the ColiGlow method, modified mTEC plate, or other methods relying on β-D-glucuronidase to differentiate *E. coli* such as Colilert and AquatTest-RUG, among others [27,37]. A possible reason for isolating microorganisms that were not *E. coli*, listed in Table 1, is that β-D-glucuronidase has been observed in other microorganisms, including many of the Enterobacteriaceae, among others [42,43]. These organisms would be capable of growing in ColiGlow media and could present as false positives if *E. coli* were also not co-located from the sample. Table 1 presents six species other than *E. coli* that were isolated, and like evaluations of Aquatest-RUG, Colilert-18, and Aquatest, *E. cloacae* and *K. pneumoniae* were isolated [27,37,44]. Other research has observed detections of *K. ascorbata* [45], *Citrobacter spp.* [46], *E. cloacae* [46], and *Klebsiella spp.* [46] from water or wastewater samples containing Colilert media and other media using similar enzymatic differentiation [47]. 

### 3.2. Tetracycline Impact on E. coli Selection and Species Identification 

In the tetracycline treated media, 28 (100%) of 28 were identified as *E. coli* versus 22 (67%) of 33 from the regular media (Table 1). There was a significant difference in the successful *E. coli* selection and identification frequency between the isolates obtained from the tetracycline-treated media versus the media without tetracycline (Fisher’s exact *p* = 0.001). The tetracycline in the media likely acted as an inhibitor or eliminated tetracycline susceptible bacteria (Figure 1), which would have included susceptible *E. coli*. Tetracycline has been used in some growth media to improve selectivity [48], and antibiotics in general are among the most used selective agents [49] possessing abilities to greatly reduce the diversity of organisms [50] limiting possible co-dependent organisms. Exploring this hypothesis, samples with fewer *E. coli* in the original source water were associated with a greater likelihood of an isolate selected that was correctly classified as *E. coli*. A plausible explanation for 100% *E. coli* recovery and isolate identification in the tetracycline-treated samples with growth is that by having less microbial diversity and a reduction in the total microbial load due to tetracycline, the treatment differentially imperiled the survival, growth, and/or selection of the non-target (non-*E. coli*) species.

### 3.3. Microbial Load and Likelihood of Selecting Non-Target Species as Isolates

When examining the relationship between the number of fluorescing ColiGlow wells (presumed to contain *E. coli*) and the likelihood of obtaining an *E. coli* isolate, the ColiGlow plates that had all 96 wells fluorescing were significantly more likely to have an isolate selected other than *E. coli* relative to the ColiGlow plates with less growth (Fisher’s Exact Test *p* = 0.005). Specifically, 91% of isolates were identified as *E. coli* when the 96-well plate did not have all 96 wells fluorescing (≤1479 MPN per 100 mL) versus 63% when all 96 wells of the ColiGlow plate were fluorescing (>1479 MPN per 100 mL). 

In comparison with other studies, using the same or similar enzyme-substrate as the ColiGlow method, the likelihood of false positives increased when non-target bacteria were in greater abundance [51,52,53,54] resulting in the recommendation [51] or actual use of substantial dilutions to enhance successful *E. coli* recovery for reducing the number of false positives [52]. When comparing the results in Table 2 with other studies, 11 (18%) of 61 isolates were not classified as *E. coli*, which if treated as false positives, would be higher than recent studies using Colilert-18, Aquatest, and Aquatest-RUG in temperate and subtropical waters that also used modified mTEC agar for colony isolation [27,44]. Specifically, those studies had false positive rates below 5%. Other studies, using original (non-modified) mTEC agar as isolation media for Colilert-18 had false positive rates of 7.4% and 36%, respectively [27,54]. 

When comparing the potential false positive rate from the ColiGlow method when the *E. coli* density estimates were within the range of the method (less than 96 wells glowing), the likelihood of recovering a non-*E. coli* species was 9%, which was closer to the false-positive rate in the more recent studies using Colilert-18 and related methods [27,44]. It is plausible that *E. coli* were also co-located in the glowing wells but were not the selected colony from the modified mTEC plates used for isolate analysis. 

### 3.4. Evaluation of Tetracycline Treatment for Screening Tetracycline-Resistant E. coli

Among 28 isolates obtained from the ColiGlow plates containing tetracycline that were evaluated for tetracycline resistance, 25 (89%) were tetracycline resistant and three (11%) were susceptible to tetracycline. When examining the three that were susceptible to tetracycline that were recovered and isolated from tetracycline-treated media, these three isolates were from ten tests that used 320 µg per 25 mL (12.8 µg/mL) versus the 640 µg per 25 mL (25.6 µg/mL). When the higher concentration was examined, 18 (100%) of 18 isolates from the higher tetracycline treatment group were tetracycline resistant versus seven (70%) of ten isolates from the treatment using half the tetracycline dose per sample, which represents a significant difference (Fisher’s Exact Test *p* = 0.037). 

Research evaluating if these antibiotic concentrations are most appropriate remains limited when examining resistance using complex environmental matrices such as contaminated surface waters. Several studies enumerating *E. coli* when tetracycline and other antibiotics have been added to Colilert were carried out from 2005 to 2010 with Ohio River (USA) surface water, [55] municipal and hospital wastewater in Ireland [56], Mud Creek (Fayetteville, Arkansas, USA) surface water impacted by wastewater effluent [57], and recently using irrigation waters on a commercial farm in Maryland, USA. [58]. The Irish study supplemented Colilert with tetracycline to achieve a concentration of 4 µg/mL and reported that 40 (100%) of 40 isolates obtained from the positive Colilert tests were tetracycline resistant. The Arkansas study used concentrations of 4, 8, and 16 µg/mL. For this research using the ColiGlow method on Kentucky waters, the lower tetracycline concentration (12.8 µg/mL) had a false-positive rate of 30% for tetracycline resistance. Differences between study methods may have contributed to the discrepancy as the Irish study used significant dilutions for ensuring samples were within the test ranges of Colilert, and by using dilutions, the dilutions reduced the complexity of the aquatic matrix which may have optimized antibiotic effectiveness. Additionally, details on the tetracycline resistance breakpoint value used in that study were not apparent, limiting comparability. 

A recent (2023) Maryland, USA, study was supplemented with 4 µg/mL (low dose) and 16 µg/mL (high dose) of tetracycline to be consistent with the Irish study [56], and current Clinical Laboratory Standards Institutes breakpoint values [59], that also aligns with the 2021 NARMS Interpretive Criteria for Susceptibility Testing [30]. In comparing low- and high-dose tetracycline treatment, significantly more growth occurred in the lower-dose samples. There was no discussion in that study on evaluating isolates for tetracycline resistance between the no dose, low-dose, and high-dose treatment groups; however, opportunities for future research were articulated.

In this study, due to the breakthrough of tetracycline susceptible organisms in the lower-dose group, the higher tetracycline concentration was subsequently used to decrease the false-positive rate. Earlier research examining tetracycline resistance in dairy cattle feces using agar-based methods that supplemented MacConkey agar with 32 µg/mL specifically used a “twofold-higher concentration” [60] of the MIC breakpoint [61]. When concentrations are used below the MIC, susceptible bacteria have been reported to survive and/or use bacterial SOS repair systems that promote horizontal gene transfer and genome mutation [62]. Accordingly, a susceptible microorganism that survived in a sub-MIC growth media could be selected and grown on the modified mTEC agar-lacking tetracycline, and then present tetracycline susceptibility when challenged by higher tetracycline concentrations in a MIC-based susceptibility analysis. Alternatively, susceptible *E. coli* bacteria surviving in sub-MIC conditions can obtain resistance genes from non-target organisms thereby having resistance induced when using sub-MIC conditions of the growth media. The sub-MIC concentrations of 1 to 15 µg/mL for tetracycline have been associated with increasing the abundance of resistance genes [63,64] which supports a perspective of attempting to maintain >15 µg/mL when evaluating tetracycline resistance among *E. coli*. Future guidelines or standards for tetracycline concentrations in screening media are recommended [15,54]. Levels in the range of >15 µg/mL [63,64] to 32 µg/mL [60] are likely needed, depending upon the density of bacteria and interfering substances in the sample. In this study, the results were mostly obtained from media containing a relatively high tetracycline concentration that would be 25.6 µg/mL when mixed with pure water, which would decrease the likelihood of sub-MIC conditions. Actual tetracycline concentrations were not measured after adding natural water samples. If measures were possible, greater understanding of how tetracycline concentrations vary overtime with samples containing varying levels of contamination may be useful for informing guidelines for media standards containing tetracycline or other antibiotics.

Overall, the use of tetracycline significantly enhanced the likelihood of isolating tetracycline-resistant *E. coli* relative to the probability of obtaining a resistant isolate from among ColiGlow plates with media that lacked tetracycline. Specifically, 25 (89%) of the 28 *E. coli* isolates from the tetracycline-treated ColiGlow plates exhibited tetracycline resistance versus 15 (32%) of the 22 *E. coli* isolates obtained from ColiGlow plates without tetracycline treatment (Chi-Square *p* < 0.001). In comparison of all isolates, including non-*E. coli* isolates, the difference in obtaining a tetracycline-resistant isolate was also significant between the tetracycline-treated and no treatment groups (Chi-Square *p* < 0.001). 

Among the 50 *E. coli* isolates evaluated in this study, the odds of observing a tetracycline-resistant *E. coli* isolate were 17.9 times higher when tetracycline was used in the media versus when it was not used (Odds Ratio = 17.9; 95% C.I.: 4.0–79.7). The area under the ROC curve was 80.7%, which is on the low end of the excellent discrimination range (80−90%) for a test [32]. Studies attempting to enumerate culturable tetracycline-resistant *E. coli* or obtain isolates would benefit from the inclusion of tetracycline in selection media. 

### 3.5. E. coli Densities and Likelihood of Obtaining Tetracycline-Resistant Isolates

Among the 61 isolates evaluated in this study, 19 were obtained from ColiGlow plates associated with a water sample that exceeded the *E. coli* density of the ColiGlow test range (>1479 MPN per 100 mL). Table 3 demonstrates that the prevalence of tetracycline resistance (84%) among the isolates associated with the highest densities of *E. coli* was significantly higher than the prevalence (55%) in isolates associated with *E. coli* densities within the range of the ColiGlow method (Fisher’s Exact Test *p =* 0.043). Among the 50 water samples that led to the successful recovery of *E. coli* isolates, 12 (100%) of the 12 isolates selected from samples associated with a ColiGlow test that exceeded the test range were resistant, which was significantly higher than the frequency of resistance among samples associated with in-range *E. coli* densities (Fisher’s Exact Test *p* = 0.002). 

Consistent with prior research, increasing *E. coli* densities were associated with a greater likelihood of observing increased tetracycline resistance among culturable *E. coli* [57], potentially driven by precipitation and runoff. Numerous studies have established relationships between increased densities of molecular markers of tetracycline resistance following precipitation events, particularly in urban and agricultural landscapes [65,66,67,68,69]. While increases in the *E. coli* densities likely increase the diversity of the *E. coli* populations which may contain antibiotic-resistant types, an increase in the overall amount of microbial, soil, and chemical contaminants could interfere with antibiotics and reduce the tetracycline exposure concentrations at the microbe level in the media to sub-MIC concentrations which could enable susceptible organisms to survive and/or induce resistance, rather than detect existing resistance, from within the sampled natural environment [60,61]. 

### 3.6. Challenges for Interpreting and Generalizing Species Identification Results

The study results are based upon a limited number of isolates (n = 61) obtained from 61 individual 96-well plates that were selected from among all the plates with presumed *E. coli* growth. Future studies evaluating recovered isolates from fecal indicator bacteria detection and enumeration media should consider examining multiple recovered isolates to strengthen the study design.

Another limitation of the study pertains to study results being aggregated from two different identification methods. While a limitation, the findings also generated additional research questions pertaining to identification methods. When limiting the species identification analyses to the results obtained by the API20E method, 30 (97%) of 31 were identified as *E. coli* which is comparable to the results reported for Aquatest-RUG (97%), modified mTEC agar with membrane filtration (97%), and Colilert-18 (98.5%) when the API20E identification method was used in related research [27]. When using the identification results from the MicroScan panels, 20 (67%) of 30 were identified as *E. coli* (Table 4). The difference in these results is significant (Fisher Exact Test *p =* 0.003) and may reflect either improved sensitivity in the MicroScan method or a significantly greater abundance of false-positive species in those samples. MicroScan methods are modern advancements relative to the API20E identification approach, and in early editions of the MicroScan methodology, the technology outperformed the API20E method by correctly identifying *E. coli* in 95% of samples versus 84% in the parallel comparison study [70]. In a recent study of isolates obtained from Colilert-18, the API20E only correctly classified 50% of the isolates from the *Enterobacter* genus [46]. The most frequent non-*E. coli* species detected in our study with the MicroScan method was *E. cloacae*, representing six (20%) of thirty isolates evaluated. *E. cloacae* was not detected in any of 31 isolates assessed by the API20E identification system, and while speculative, this organism may have been incorrectly classified as *E. coli* in the API20E tests; however, no side-by-side comparison was completed.

Beyond MicroScan and API20E identification methods, molecular or MALDI-TOF approaches are promising. Molecular approaches targeting and/or quantifying the *uidA* may have a similar false-positive detection as experienced in culture-based assays as the *uidA* gene encodes for beta-glucuronidase [38] which is responsible for the fluorescence observed in this study and other enzyme-substrate tests such as Colilert. The *uidA* gene can exist in *E. cloacae*, *K. pneumonia*, and other coliforms [40,71]. For improving identification, multiplex PCRs [71], as well as 16S rRNA and/or MALDI-TOF approaches have been recommended due to their high accuracy and reliability [72].

### 3.7. Value of Applying Tetracycline-Treated Culture-Based E. coli Detection Methods

The use of tetracycline in this study reduced the total number of tetracycline-susceptible *E. coli* and other susceptible bacteria. The three-year study that led to the study of 61 isolates included 224 ColiGlow plates, which included 112 plates containing media treated with tetracycline. Among these ColiGlow plates with tetracycline in the media, 63 (56%) of the 112 samples had no growth. Conversely, when using the regular ColiGlow method, where tetracycline was not in the media, only seven (6.25%) of those one hundred and twelve samples had no detectable *E. coli* growth. Together, these results indicate that when tetracycline was used in the media, there was a significant reduction in the likelihood of observing any *E. coli* growth (Chi-Square *p* < 0.001) by presumably eliminating or inhibiting the growth and/or enzymatic expression among the tetracycline susceptible *E. coli*. Using this information, along with the confirmation of tetracycline resistance in nearly all isolates when tetracycline was used, these data support the value of the ColiGlow method with tetracycline for screening and detecting tetracycline-resistant *E. coli* from water samples when the samples have *E. coli* densities within the range of the method. When water samples are expected to exceed the range, such as following wet weather events, the use of dilutions would likely improve the classifications for true *E. coli* and tetracycline resistance [52].

Practical methods for enumerating tetracycline-resistant *E. coli* may enhance local surveillance in limited resource settings for similar reasons needed for global water quality monitoring using total *E. coli*. Specifically, regions experiencing the greatest threats to water safety stand to benefit from low-cost and field-ready methods that can be employed [17,18,27], and limited resource environments have substantial overlap with areas experiencing the greatest amount of pressure from waterborne illness, mortality, and emerging antibiotic resistance [1,3,4,5,7]. Additional environments with limited laboratory resources may include agricultural areas where irrigation waters and livestock-impacted waters could also be actively monitored [58].

In addition to the practicality of enumeration methods, culturable *E. coli* and tetracycline-resistant *E. coli* enable the selection of *E. coli* isolates that can be studied, further enabling a comprehensive and simultaneous characterization of susceptibility to multiple antibiotics beyond tetracycline, while also enabling molecular approaches to examine or identify resistance genes specific to these isolates [73]. Ultimately, approaches using molecular- and/or culture-based methods are needed and valued for environmental AMR surveillance and the value of each remains dependent on the purpose of the surveillance [73,74]. Research applications using existing methods for quantifying both total *E. coli* and tetracycline-resistant *E. coli* densities to obtain the percentages of resistant *E. coli* have been proposed for informing how water quality parameters or contaminants may influence the relative abundance of tetracycline-resistant *E. coli* versus total *E. coli* [58].

## 4. Conclusions

The odds of isolating a tetracycline-resistant *E. coli* increased nearly 18-fold when tetracycline was used in the ColiGlow method relative to when tetracycline was not added. The addition of tetracycline to the media at the concentrations used in this study resulted in 100% of the *E. coli* isolates also being observed as tetracycline-resistant when the total *E. coli* density in the sample water was within the range of the enumeration method (<1479 MPN per 100 mL). The successful recovery of *E. coli* isolates and tetracycline-resistant isolates diminished when samples had elevated densities of *E. coli* and other enteric bacteria that also express β-D-glucuronidase activity. The use of dilutions aimed at achieving densities within the enumeration range of the method may enhance the discrimination of the method for correctly classifying *E. coli* and tetracycline resistance. The species identification methods used in this study may have led to misclassification and future studies aimed at identifying bacteria isolated from media using β-D-glucuronidase enzymatic should consider MALDI-TOF or molecular identification methods. Overall, these results demonstrate that the ColiGlow method, with and without tetracycline, has acceptable discrimination for identifying *E. coli* and tetracycline-resistant *E. coli*, respectively. In tandem, these methods and related methods have value for surveillance efforts aimed at understanding how environmental factors influence the densities and spatiotemporal distributions of tetracycline-resistant culturable *E. coli* in natural waters.

## Figures and Tables

**Table 1:** The species and tetracycline susceptibility status of microorganisms isolated in this study from fluorescing ColiGlow wells from growth media with and without tetracycline.

	Isolates from ColiGlow Wells without Tetracycline		Isolates from ColiGlow Wells with Tetracycline	
Species (no.)	Susceptible	Resistant	Susceptible	Resistant
*Escherichia coli* (50)	15	7	3	25
*Enterobacter cloacae* (6)	2	4	0	0
*Kluyvera ascorbate* (1)	0	1	0	0
*Kluyvera intermedia* (1)	1	0	0	0
*Klebsiella pneumoniae* (1)	0	1	0	0
*Serratia odorifera* (1)	0	1	0	0
*Citrobacter braakii* (1)	1	0	0	0
Total (61)	19	14	3	25

**Table 2:** The species and tetracycline susceptibility status of microorganisms isolated in this study from fluorescing ColiGlow wells from growth media with and without tetracycline.

Species Identification Reported by ID Method	Frequency (%)	
	*E. coli* Density within Method Range	*E. coli* Density over Method Range
*E. coli*	38 (90.5%)	12 (63.2%)
Not *E. coli*	4 (9.5%)	7 (37.8%)
Overall	42 (100%)	19 (100%)

**Table 3:** The tetracycline susceptibility status of microorganisms isolated in this study from fluorescing ColiGlow wells when *E. coli* densities are in-range or over-range (>1479 MPN per 100 mL) in the water sample.

Tetracycline Susceptibility	Frequency (%)	
	*E. coli* Density within Method Range	*E. coli* Density over Method Range
Tetracycline-Resistant	23 (54.8%)	16 (84.2%)
Tetracycline-Susceptible	19 (45.2%)	3 (15.8%)
Overall: All Isolates	42 (100.0%)	19 (100.0%)
Tetracycline-Resistant *E. coli*	18 (52.6%)	12 (100.0%)
Tetracycline-Susceptible *E. coli*	20 (47.4%)	0 (0%)
Overall: *E. coli* Isolates	38 (100.0%)	12 (100.0%)

**Table 4:** The frequency of 31 isolates reported as *E. coli* or not *E. coli* by the API20E identification method versus the frequency reported as *E. coli* or not *E. coli* for 30 different isolates using the MicroScan instrument.

Species Identification Reported	Frequency (%)	
	API20E Identification Method	MicroScan Urine Panel-85 Identification Method
*E. coli*	30 (96.8%)	20 (66.7%)
Not *E. coli*	1 (3.2%)	10 (33.3%)
Overall	31 (100%)	30 (100%)

## Data Availability

Raw data available upon request.
